# Do presenting symptoms, use of pre-diagnostic endoscopy and risk of emergency cancer diagnosis vary by comorbidity burden and type in patients with colorectal cancer?

**DOI:** 10.1038/s41416-021-01603-7

**Published:** 2021-11-05

**Authors:** Sara Benitez Majano, Georgios Lyratzopoulos, Bernard Rachet, Niek J. de Wit, Cristina Renzi

**Affiliations:** 1 Inequalities in Cancer Outcomes Network (ICON) Group, Department of Non-communicable Disease Epidemiology, London School of Hygiene and Tropical Medicine, Keppel St, Bloomsbury, London, WC1E 7HT UK; 2 Epidemiology of Cancer Healthcare & Outcomes (ECHO) Research Group, Department of Behavioural Science and Health, Institute of Epidemiology & Health Care, University College London, London, WC1E 7HB UK; 3 University Medical Center, Utrecht University, Julius Center for Health Sciences and Primary Care, PO Box 85500, 3508 GA Utrecht, The Netherlands

**Keywords:** Diagnosis, Endoscopy, Comorbidities, Epidemiology, Colon cancer

## Abstract

**Background:**

Cancer patients often have pre-existing comorbidities, which can influence timeliness of cancer diagnosis. We examined symptoms, investigations and emergency presentation (EP) risk among colorectal cancer (CRC) patients by comorbidity status.

**Methods:**

Using linked cancer registration, primary care and hospital records of 4836 CRC patients (2011–2015), and multivariate quantile and logistic regression, we examined variations in specialist investigations, diagnostic intervals and EP risk.

**Results:**

Among colon cancer patients, 46% had at least one pre-existing hospital-recorded comorbidity, most frequently cardiovascular disease (CVD, 18%). Comorbid versus non-comorbid cancer patients more frequently had records of anaemia (43% vs 38%), less frequently rectal bleeding/change in bowel habit (20% vs 27%), and longer intervals from symptom-to-first relevant test (median 136 vs 74 days). Comorbid patients were less likely investigated with colonoscopy/sigmoidoscopy, independently of symptoms (adjusted OR = 0.7[0.6, 0.9] for Charlson comorbidity score 1–2 and OR = 0.5 [0.4–0.7] for score 3+ versus 0. EP risk increased with comorbidity score 0, 1, 2, 3+: 23%, 35%, 33%, 47%; adjusted OR = 1.8 [1.4, 2.2]; 1.7 [1.3, 2.3]; 3.0 [2.3, 4.0]) and for patients with CVD (adjusted OR = 2.0 [1.5, 2.5]).

**Conclusions:**

Comorbid individuals with as-yet-undiagnosed CRC often present with general rather than localising symptoms and are less likely promptly investigated with colonoscopy/sigmoidoscopy. Comorbidity is a risk factor for diagnostic delay and has potential, additionally to symptoms, as risk-stratifier for prioritising patients needing prompt assessment to reduce EP.

## Background

Diagnosing colorectal cancer (CRC) at an early stage and before it becomes a medical emergency is paramount for improving survival, patient reported outcomes and disruptions to health services [1–3]. Efforts to improve the timely diagnosis of cancer in England have included the roll out of a population screening programme since 2006, fast-track diagnostic pathways for patients with symptoms suggestive of cancer and recommendations on the use faecal immunochemical testing (FIT) in primary care for patients with low-risk bowel symptoms to guide specialist referrals [4–6]. In spite of improvements, large proportions of patients are still diagnosed following an emergency admission and/or with late stage disease, having substantially poorer outcomes than those diagnosed through non-emergency routes and/or with early-stage disease [3, 4]. In England 23% of CRC are diagnosed following an emergency presentation, [3] with international figures ranging between 11 and 39% [7]. Emergency diagnosis is associated with advanced cancer stage and poorer survival, even after controlling for stage [7]. One-year survival is 49% after emergency CRC diagnosis compared to over 80% for non-emergency routes [3]. Emergency presentations are associated with worse patient-reported outcomes, disruptions to hospital services [2, 8, 9] and affect healthcare resource use [10]. Reducing emergency presentations is therefore considered a key public health target [1].

The relevance of pre-existing conditions (comorbidities) in influencing the timely diagnosis of cancer and cancer outcomes has been increasingly recognised [11–17]. The COVID-19 pandemic, during which many cancer investigations have been postponed [18–20], with an expected 17% increase in CRC deaths, [18] has highlighted the urgent need of identifying patient groups at higher risk of diagnostic delays, such as those with non-specific symptoms and/or comorbidities [19–21]. Previous research has shown that many patients with comorbidities repeatedly present to their doctor with cancer-related symptoms in the two years before being diagnosed with cancer as an emergency [22]. However, little is known on how comorbidities may affect different aspects of the diagnostic process, such as presenting symptoms, timeliness of investigations and length of diagnostic intervals [11].

To enhance risk-stratification approaches and improve diagnostic timeliness and cancer outcomes, we need to understand likely differences in symptomatic presentations and use of diagnostic investigations by morbidity status. We aimed to evaluate the association between pre-existing morbidities, and symptomatic presentation, the use of diagnostic investigations and the risk of emergency diagnosis among symptomatic patients subsequently diagnosed with CRC. We focused on patients presenting with possible cancer symptoms as they represent the great majority of CRC cases (over 90%) and they have worse outcomes than screening-detected individuals [3, 23, 24]. The ultimate aim is to provide evidence for informing decision-making regarding referrals/diagnostic investigations for symptomatic patients.

## Methods

### Study population and data sources

We performed a cohort study on patients aged 18–99 years diagnosed with colon cancer (International Classification of Diseases 10th Edition, ICD-10, codes C18.1–C18.9) or rectal cancer (ICD-10 codes C19–C20) using National Cancer Registry records, linked to primary care data from the Clinical Practice Research Datalink (CPRD). The Cancer Registry includes all cancers diagnosed in England, with excellent completeness and high quality data [25]. CPRD has data for over 11 million patients from more than 670 general practices across the United Kingdom [26] and is representative of the general population [27]. It provides prospectively collected patient-level information on signs/symptoms, diagnoses and tests. In addition, we obtained individually linked secondary care records from the Hospital Episode Statistics (HES) on outpatient and admitted patient care. Information on the cancer site, date of diagnosis and age at diagnosis was obtained from the linked National Cancer Registry. Data linkage is carried out by the Trusted Third Party NHS Digital following high standards [28].

To be included, patients had to be diagnosed between 2011 and 2015 and have at least one new-onset sign/symptom potentially related to CRC (Supplementary code-list Box 1) recorded in primary care pre-cancer diagnosis. We concentrated on new-onset symptoms, which are more likely associated with an as-yet undiagnosed cancer, rather than chronic symptoms, which are usually due to long-standing benign conditions (e.g. diverticular disease or irritable bowel syndrome). New-onset symptoms were defined as symptom recorded for the first time during the 2 years pre-cancer diagnosis, with no prior record of the same symptom during the 3–5 years pre-cancer.

**Fig. 1:**
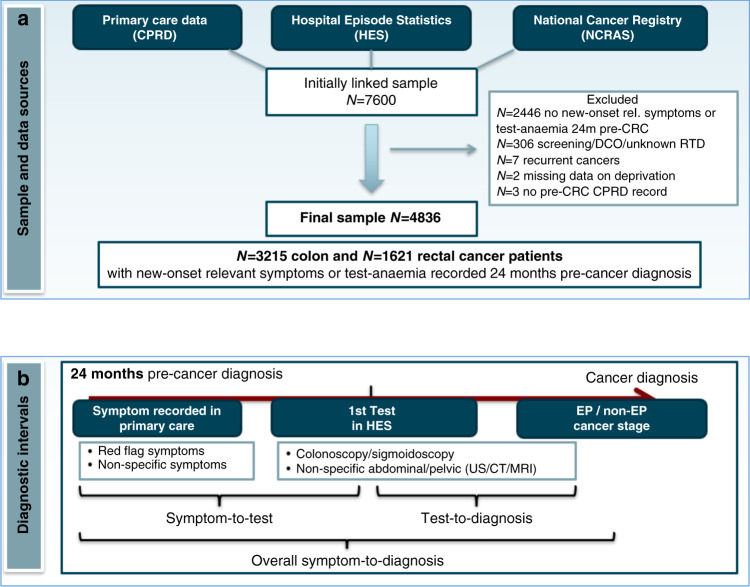
Study population, data sources and diagnostic intervals from symptomatic presentation to cancer diagnosis. **a** Study population and data sources. **b** Diagnostic intervals from symptomatic primary care presentation to cancer diagnosis.

As the study focused on symptomatic patients, we excluded patients whose diagnostic route was classified as screening (Fig. 1a). The final study sample comprised a total of 4836 cancer patients, which is in line with expectations, considering that CPRD covers 7% of the population in England [27].

### Study variables

The main explanatory variables were comorbidities recorded in HES during the 6 years before and up to the CRC diagnosis. We focused on hospital-recorded comorbidities to provide evidence on the likely role played by severe morbidities in influencing the use and timeliness of diagnostic endoscopy, due to their possible impact on patients’ procedural risk. In addition to examining the most common severe morbidities in hospitalised patients, we obtained two further measures of comorbidity burden, the Charlson comorbidity score and the count of morbidities. Using a validated algorithm [29], we identified morbidities through ICD-10 codes within HES in-patient and out-patient records and selected the 17 conditions typically included in the Charlson score (Box 1). We used the Charlson score as the main measure of comorbidity burden, as it is likely to better reflect the assessment of procedural risk than the morbidity count.

Further explanatory variables were red flag symptoms/signs recorded in primary care pre-cancer diagnosis, including CRC-localising symptoms (rectal bleeding or change in bowel habit) and non-localising signs (laboratory-confirmed anaemia); we also examined non-red flag symptoms potentially related to CRC (including abdominal pain, constipation, diarrhoea, weight loss and fatigue) [30, 31]. Classification in red flag or non-red flag symptoms follows the UK NICE guidelines for suspected cancer recognition and referral [31]. Change in bowel habit, as well as rectal bleeding or unexplained anaemia, are considered red flag symptoms, which warrant urgent referral for investigations, as they have a Positive Predictive Value (PPV) >3% for colorectal cancer. Other symptoms, such as diarrhea, constipation or abdominal pain are associated with a lower PPV for colorectal cancer and are not considered as red flag symptoms based on NICE guidelines. While the terms ‘diarrhea’ or ‘constipation’ can be considered as deviations from normal bowel patterns, the term ‘change in bowel habit’ is typically used in UK primary care records when there is a higher suspicion of CRC, as it warrants urgent referral according to NICE guidelines.

Relevant symptoms/signs (and related Medcodes/Readcodes) have been defined based on the literature, guidelines [31] and clinical expert revisions and classified in new-onset symptoms, if recorded for the first time during the 2 years pre-cancer diagnosis, with no prior record of the same symptoms 3–5 years prior. Anaemia was defined based on haemoglobin-tests with values below the normal gender-specific range provided by CPRD. Socio-demographic characteristics included gender, age and deprivation (Index of Multiple Deprivation for England). Information on TNM grouping stage at diagnosis was available from the linked National Cancer Registry.

Information on diagnostic investigations was extracted from in-patient and out-patient HES records in the 2 years pre-cancer diagnoses using OPCS 4.5 Standard Classification for NHS procedures codes (Supplementary code-list Box 2). Investigations were classified as colonoscopy/flexible sigmoidoscopy and non-specific abdominal/pelvic tests (abdominal ultrasound, abdominal CT/MRI, gastroscopy, small bowel endoscopy, gynaecologic tests, pelvic MRI/CT and pelvic ultrasound). To be included in the analysis, investigations must have occurred following a CRC-relevant symptom recorded in primary care. Investigations, such as chest x-rays or cardiac tests, which might have been requested for other reasons (e.g. respiratory or cardiac symptoms) were excluded a priori to minimise bias.

We calculated the ‘symptom-to-test’ interval as the time (in days) from the first new-onset symptom recorded in primary care during the 24 months pre-cancer diagnosis to the first HES-recorded investigation. The date of the first relevant symptom recording typically corresponds to the primary care consultation date, as CPRD includes prospectively collected information on symptoms recorded at the time of consultation. The ‘test-to-diagnosis’ interval was the time from the first investigation to cancer diagnosis. We also estimated the ‘overall symptom-to-diagnosis’ interval from the first new-onset relevant symptom during the 24 months pre-cancer to cancer diagnosis (Fig. 1b). The date of diagnosis was based on the data provided by the National Cancer Registry, which follows the rules of the European Network of Cancer Registries [25].

Additionally, we performed sensitivity analyses focusing only on the timing of bowel endoscopy (independently if this was the first test), as this is very well documented in HES [12, 32]. Previous validation studies have shown 96% accuracy for routine data on investigations collected in HES [32, 33]. This sensitivity analysis excluded 1398 patients without evidence of colonoscopy/sigmoidoscopy and focused on the 3436 patients with lower GI endoscopy, among which 73% had an endoscopy as the first and only investigation, 18% as first investigation followed by other tests and 9% as second investigation preceded by non-specific abdominal/pelvic tests.

Information on emergency cancer diagnosis was derived from the linked Cancer Registry and defined according to the Routes to Diagnosis algorithm (Box 2), i.e. diagnosis following presentation to Accident and Emergency, GP emergency referrals, or emergency pathways for in/out-patients [3, 23].

Box 1 Comorbidities included in the study (following the Charlson comorbidity index definition and selected using a validated algorithm [29]) Myocardial infarction

Congestive heart failure

Peripheral vascular disease

Cerebrovascular disease

Dementia

COPD

Rheumatic disease

Peptic ulcer disease

Mild liver disease

Diabetes without chronic complications

Diabetes with chronic complications

Hemiplegia or paraplegia

Renal disease

Moderate or severe liver disease

AIDS/HIV

Any malignancy

Metastatic solid tumours

Box 2 Routes to diagnosis for symptomatic patients included in the study (based on NCIN-PHE) [23, 66] **Emergency cancer diagnosis**

- Diagnosis of cancer following presentation to an Accident and Emergency Unit or a GP emergency referral or emergency pathways for in/out-patients.

**Non-emergency cancer diagnoses**

- ***Two-week wait referrals***: Patients referred urgently by their GP for suspected cancer, so that they can see a specialist within 2 weeks (introduced in England in 2000).
- ***Routine GP referrals***: Patients referred by their GP but not under the two-week wait referral route.
- ***Elective outpatient/inpatient***: An elective route starting with an outpatient appointment, either consultant to consultant referral or other referral; or starting with inpatient admission where no earlier information is available from waiting list prior to admission.

### Statistical analysis

Initially we described socio-demographic and clinical characteristics of patients, including comorbidities, symptoms, type of investigations, stage at diagnosis, and emergency presentation status. We used Chi-squared tests to compare the distribution of socio-demographic and clinical characteristics by comorbidity status.

We then performed two multivariable analyses. In the first, we employed quantile multivariate regression to assess the variation in diagnostic intervals (time intervals between symptomatic presentation in primary care and the use of relevant diagnostic investigations) by comorbidity and symptoms, accounting for age, sex and deprivation. Quantile regression is an extension of linear regression for skewed data and is a well-established approach for examining variation in time intervals to test, diagnosis or treatment all of which are known to be right-skewed [34–37]. It allowed us to compare diagnostic intervals by comorbidity status and other patient characteristics at different centiles (50, 75 and 90) of the diagnostic intervals. We focused on the median (50th centile) and 75th centile. The final quantile regression model for each diagnostic interval included variables a priori deemed relevant for interval length, based on the literature and clinical reasoning (morbidities, symptoms, socio-demographic factors). We focused on the interval length and used quantile regression, rather than hazard models, as interval length is a more intuitive outcome in this study: expressing how each variable affects the interval(s) in absolute terms is preferable from a clinical and public health perspective, rather than reporting the findings in relative terms, as would be the case with hazard models.

In the second analysis, we used multivariable logistic regression to assess the risk of emergency cancer diagnosis by comorbidity burden/status, accounting for symptoms, type of first investigation and socio-demographic characteristics. The final model encompassed all variables thought a priori to be potentially relevant in influencing the risk of emergency presentations based on the literature and clinical reasoning. We also examined associations between the likelihood of having a colonoscopy/flexible sigmoidoscopy as first investigation and comorbidity burden/status, symptoms and socio-demographic characteristics.

As general practices can vary substantially, for example in their level of use of investigations such as endoscopies [38–40], the analyses accounted for patient clustering by practice and estimated robust standard errors. The multivariable models did not include variables potentially on the causal pathway (mediators): diagnostic route and type of first investigation, as they might be between comorbidity, symptomatic presentation, and length of diagnostic intervals (for example, colonoscopy is diagnostic in most cases [41]); stage at diagnosis, as it might be on the causal pathway between comorbidity and emergency presentation. Furthermore, to reduce possible bias, we used different multivariable models, each with a different morbidity measure (Charlson score, morbidity count, specific morbidity variables), as these variables were a priori highly correlated by construction, i.e. one is partly embedded in another. Finally, to assess how the odds ratios change when accounting for covariates, we additionally present the findings from the unadjusted models, as well as of the models including only socio-demographic factors, followed by comorbidities, then symptoms and investigations. We used Stata 15 for all statistical analyses.

## Results

Among the 3,215 symptomatic colon and 1621 rectal cancer patients, approximately 46% and 36%, respectively, had at least one morbidity recorded in HES during the 6 years pre-cancer diagnosis, while 20% and 14%, respectively, had two or more morbidities (multimorbidity) (Table 1). The most common morbidities, included cardiovascular (CVD), chronic obstructive pulmonary disease (COPD) and diabetes, both among colon (18%, 17%, 15%, respectively) and rectal cancer patients (13%, 13%, 12%, respectively).

**Table 1:** Patient characteristics, diagnostic investigations and routes to cancer diagnosis by comorbidity status.

	Colon				Rectum			
	All patients	Non comorbid	Comorbid	Chi2 *p* value	All patients	Non comorbid	Comorbid	Chi2 *p* value
*Age group*				<0.001				<0.001
<45	83 (2.6)	71 (4.1)	12 (0.8)		48 (3.0)	44 (4.2)	4 (0.7)	
45–54	235 (7.3)	182 (10.5)	53 (3.6)		173 (10.7)	150 (14.4)	23 (4.0)	
55–64	479 (14.9)	320 (18.5)	159 (10.7)		327 (20.2)	248 (23.9)	79 (13.6)	
65–74	762 (23.7)	433 (25.0)	329 (22.2)		422 (26.0)	277 (26.7)	145 (24.9)	
75–84	1144 (35.6)	513 (29.7)	631 (42.5)		473 (29.2)	251 (24.2)	222 (38.1)	
85+	512 (15.9)	211 (12.2)	301 (20.3)		178 (11.0)	69 (6.6)	109 (18.7)	
*Sex*				0.132				0.131
Male	1569 (48.8)	823 (47.6)	746 (50.2)		974 (60.1)	610 (58.7)	364 (62.5)	
Female	1646 (51.2)	907 (52.4)	739 (49.8)		647 (39.9)	429 (41.3)	218 (37.5)	
*Deprivation quintile*				<0.001				0.008
1 (Least deprived)	808 (25.1)	457 (26.4)	351 (23.6)		379 (23.4)	258 (24.8)	121 (20.8)	
2	745 (23.2)	406 (23.5)	339 (22.8)		348 (21.5)	220 (21.2)	128 (22.0)	
3	684 (21.3)	395 (22.8)	289 (19.5)		362 (22.3)	242 (23.3)	120 (20.6)	
4	557 (17.3)	286 (16.5)	271 (18.2)		283 (17.5)	183 (17.6)	100 (17.2)	
5 (most deprived)	421 (13.1)	186 (10.8)	235 (15.8)		249 (15.4)	136 (13.1)	113 (19.4)	
*New-onset symptoms in the two years before CRC diagnosis*								
Rectal bleeding or CIBH	767 (23.9)	465 (26.9)	302 (20.3)	<0.001	1034 (63.8)	730 (70.3)	304 (52.2)	<0.001
Anaemia (Lab-based)	1285 (40.0)	650 (37.6)	635 (42.8)		272 (16.8)	122 (11.7)	150 (25.8)	
Non red-flag symptoms	1163 (36.2)	615 (35.5)	548 (36.9)		315 (19.4)	187 (18.0)	128 (22.0)	
*Had Colonoscopy/Flexible Sigmoidoscopy two years before CRC diagnosis*				<0.001				0.013
No	1143 (35.6)	550 (31.8)	593 (39.9)		255 (15.7)	146 (14.1)	109 (18.7)	
Yes	2072 (64.4)	1180 (68.2)	892 (60.1)		1366 (84.3)	893 (85.9)	473 (81.3)	
*Type of first investigation in the two years prior CRC diagnosis*^*a*^				<0.001				<0.001
Bowel endoscopy	1713 (53.3)	1022 (59.1)	691 (46.5)		1276 (78.7)	860 (82.8)	416 (71.5)	
Abdominal/Pelvic	588 (18.3)	247 (14.3)	341 (23.0)		114 (7.0)	36 (3.5)	78 (13.4)	
Non-specific abdominal	914 (28.4)	461 (26.6)	453 (30.5)		231 (14.3)	143 (13.8)	88 (15.1)	
*Route to diagnosis*				<0.001				<0.001
Emergency presentation	957 (29.8)	401 (23.2)	556 (37.4)		185 (11.4)	75 (7.2)	110 (18.9)	
Two-week wait	1086 (33.8)	668 (38.6)	418 (28.1)		786 (48.5)	556 (53.5)	230 (39.5)	
GP referral	806 (25.1)	459 (26.5)	347 (23.4)		490 (30.2)	315 (30.3)	175 (30.1)	
Other in/outpatient	366 (11.4)	202 (11.7)	164 (11.0)		160 (9.9)	93 (9.0)	67 (11.5)	
*TNM Stage at diagnosis*^b^				0.637				0.107
I	268 (10.6)	156 (11.2)	112 (9.9)		251 (20.4)	162 (19.8)	89 (21.8)	
II	778 (30.8)	424 (30.4)	354 (31.3)		264 (21.5)	171 (20.9)	93 (22.7)	
III	707 (28.0)	397 (28.4)	310 (27.4)		421 (34.3)	300 (36.6)	121 (29.6)	
IV	773 (30.6)	419 (30.0)	354 (31.3)		292 (23.8)	186 (22.7)	106 (25.9)	
Missing	689 (21.4)	334 (19.3)	355 (23.9)		393 (24.2)	220 (21.2)	173 (29.7)	
*Specific comorbidities*								
CVD	583 (18.1)				209 (12.9)			
COPD	560 (17.4)				217 (13.4)			
Diabetes	493 (15.3)				201 (12.4)			
Renal disease	224 (7.0)				91 (5.6)			
*Comorbidity count*								
0	1730 (53.8)				1039 (64.1)			
1	840 (26.1)				357 (22.0)			
2	384 (11.9)				127 (7.8)			
3+	261 (8.1)				98 (6.0)			
*Charlson Comorbidity Index (CCI) score*								
0	1730 (53.8)				1039 (64.1)			
1	750 (23.3)				315 (19.4)			
2	343 (10.7)				124 (7.6)			
3+	392 (12.2)				143 (8.8)			
*Total*	3215 (100.0)	1730 (100.0)	1485 (100.0)		1621 (100.0)	1039 (100.0)	582 (100.0)	

*CIBH* change in bowel habit; non red-flag symptoms include abdominal pain, constipation, diarrhoea, weight loss and fatigue; *CVD* cardiovascular disease, *COPD* chronic obstructive pulmonary disease.

^a^A bowel endoscopy was performed following non-specific abdominal/pelvic tests (rather than as first test) in 11% of colon and 5% of rectal cancer patients.

^b^Stage I–IV percentages refer to the subgroup of patients with stage information. Percentage of missing stage refers to the total sample.

Overall, 64% of colon and 81% of rectal cancer patients had at least one red flag sign/symptom (anaemia, rectal bleeding or change in bowel habit) recorded in the two years pre-cancer diagnosis; 36 and 19% had non-red flag relevant symptoms only (including abdominal pain, weight loss, fatigue). The single most common relevant sign/symptom was lab-based anaemia in colon cancer (40%) and rectal bleeding in rectal cancer patients (46%).

A colonoscopy or flexible sigmoidoscopy was performed as first test to investigate relevant symptoms in 53% of colon and 79% of rectal cancer patients. In a small proportion of patients (11 and 6%, respectively) a colonoscopy/sigmoidoscopy followed non-specific abdominal/pelvic tests. Overall, the most frequent route to cancer diagnosis was the urgent 2-week wait (TWW) referral, but emergency diagnoses occurred in 30% of colon and 11% of rectal cancer patients. Information on stage at diagnosis was available for 78% of patients and, among them, stage IV disease accounted for 31% of colon and 24% of rectal cancers (Table 1).

### Comparison of patient characteristics, investigation use and diagnostic route by comorbidity burden/status

Patients with at least one hospital-recorded comorbidity, compared to those without, tended to be older and more deprived; they more frequently had a record of anaemia and less frequently rectal bleeding or change in bowel habit (Table 1). Similarly, patients with the most common specific morbidities (CVD, COPD, diabetes), versus patients without such conditions, more frequently had records of anaemia and less frequently rectal bleeding or change in bowel habit. This was particularly marked among rectal cancer patients, where anaemia was recorded in 31% of CVD patients, 28% in COPD and 26% in diabetic patients, versus 15% in patients without any of these conditions; conversely, rectal bleeding/change in bowel habit was recorded in 49% of CVD patients, 49% of COPD and 51% of diabetic patients, versus 66% of patients without any such conditions (*p* < 0.001). A haemoglobin-test was performed more frequently in comorbid versus non-comorbid patients during the 24 months pre-cancer diagnosis (colon cancer: 93% versus 88%; rectal cancer: 91% versus 82%). In those who were tested, anaemia prevalence in the 24 months pre-cancer was higher in comorbid versus non-comorbid patients (colon cancer: 52% versus 48%; rectal cancer: 36% versus 22%).

Examining the type of first abdominal/pelvic investigation used after patients presented with CRC-relevant symptoms shows that colonoscopy/sigmoidoscopy was used less frequently in comorbid versus non-comorbid patients (colon cancer: 47% versus 59%; rectal cancer: 72% versus 83%). This was confirmed at multivariable analyses accounting for symptoms and patient characteristics: the likelihood of being investigated with a colonoscopy/flexible sigmoidoscopy was significantly lower for patients with Charlson comorbidity score 1, 2, 3+ versus 0 (adjusted (a)OR = 0.7 [95% CI 0.6–0.9]; 0.7 [0.6–0.9]; 0.5 [0.4–0.7], respectively); there was no variation by type of endoscopy, i.e. having a comorbidity was associated with a lower likelihood of colonoscopy, as well as of sigmoidoscopy as first test (data not shown). Examining the most common specific morbidities showed similar findings, for example among colon cancer patients, the likelihood of endoscopy was lower for patients with versus without CVD (44% versus 55%; aOR=0.7 [95% CI 0.6–0.9] and for those with versus without renal disease (38% versus 54%; aOR=0.7 [95% CI 0.5–0.9].

Comorbid versus non-comorbid patients with CRC-related symptoms were diagnosed more frequently with cancer through the emergency route (colon cancer: 37% versus 23%; rectal cancer: 19% versus 7%, respectively) and less frequently through the expedited two-week wait route (Table 1). Similarly, patients with CVD or with chronic renal disease were more frequently diagnosed with colon cancer through the emergency route compared to those without these conditions (CVD: 43% versus 27%; renal disease: 45% versus 29%).

### Association between patient characteristics, symptoms, comorbidity burden/status and length of diagnostic intervals

**Fig. 2:**
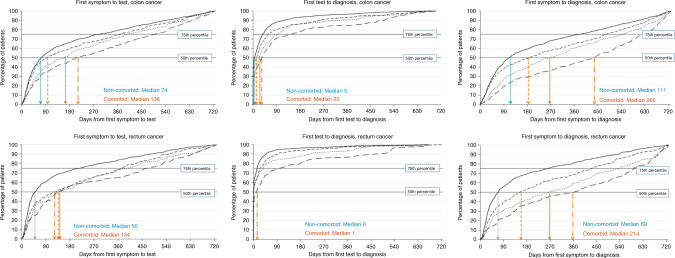
Time intervals (days) from symptomatic primary care presentation to cancer diagnosis. Values are shown for colon (top row) and rectal cancer (bottom row) patients by Charlson comorbidity Index (CCI) score. Solid line CCI score 0; dashed line CCI score 1; dotted line CCI score 2; large dashed line CCI score 3+. Symptom to test and test to diagnosis intervals refer to the subgroup of patients with a relevant test (*n* = 2301 colon; *n* = 1390 rectum), while the symptom to diagnosis intervals refer to the total sample (*n* = 3215 colon; *n* = 1621 rectum). The symptom-to-diagnosis interval for the subgroup of tested individuals is shown Supplementary Fig. 1. Note: The median values shown here are statistically significantly different (*p* < 0.001) when comparing patients with Charlson score 1+ versus 0.

The symptom-to-test, test-to-diagnosis and overall symptom-to-diagnosis intervals were notably longer for comorbid versus non-comorbid patients: median 136 versus 74 days; 20 versus 5 days; and 266 versus 111 days, respectively, among colon cancer patients (Fig. 2 and Table 2; Supplementary Tables 1–2; Supplementary Fig. 1). Further, diagnostic intervals increased with increasing comorbidity burden (Fig. 2 and Supplementary Table 2) and were longer in patients with the most common individual morbidities compared to those without such morbidities: e.g. the longest symptom-to-test, test-to-diagnosis and overall symptom-to-diagnosis intervals were observed for patients with chronic renal disease versus those without: median 201 versus 88 days; 38 versus 10 days; 432 versus 151 days, respectively, among colon cancer patients; and for patients with diabetes versus those without: median 188 versus 85 days; 17 versus 10 days; 344 versus 140 days, respectively (Supplementary Table 3).

**Table 2:** Interval (in days) from first symptom to first investigation, descriptive statistics and multivariate quantile regression (*n* = 3215 colon; *n* = 1621 rectum).

	Colon cancer									Rectal cancer								
	Percentiles			COLON Multivariate quantile regression						Percentiles			RECTUM Multivariate quantile regression					
	50th	75th	90th	50th percentile			75th percentile			50th	75th	90th	50th percentile			75th percentile		
				Adj. interval	95% CI	*p*-value	Adj. interval	95% CI	*p*-value				Adj. interval	95% CI	*p*-value	Adj. interval	95% CI	*p*-value
*Reference group*^a^				29.7	1.7, 44.7		97.3	49.4, 150.6					38.0	19.8, 56.2		155.0	65.0, 245.0	
*Age group*																		
<45	131	348	666	71.5	−56.3, 199.4	0.373	234.7	74.0, 395.4	0.017	43	108	386	32.0	−12.7, 76.7	0.657	63.0	−136.1, 262.1	0.098
45–54	88	321	558	40.5	−9.5, 90.6	0.234	178.3	6.6, 350.0	0.205	63	257	506	47.5	8.2, 86.8	0.377	175.0	−46.1, 396.1	0.765
55–64	65	237	512							58	259	498						
65–74	71	306	541	23.7	−16.9, 64.3	0.959	155.0	37.6, 272.4	0.106	55	211	444	32.0	−1.4, 65.4	0.438	97.0	−77.1, 271.1	0.176
75–84	112	401	611	58.7	13.9, 103.5	0.003	240.7	125.9, 355.4	<0.001	106	376	566	63.5	12.6, 114.4	0.127	225.0	40.4, 409.6	0.147
85+	176	457	626	107.0	32.4, 181.6	0.002	263.0	127.9, 398.1	<0.001	140	433	581	53.0	−20.3, 126.3	0.594	233.0	38.8, 427.2	0.142
*Sex*																		
Male	87	337	561							66	266	520						
Female	108	374	599	25.7	−11.1, 62.5	0.749	128.3	35.7, 221.0	0.187	79	342	551	57.0	22.2, 91.8	0.025	199.0	60.6, 337.4	0.075
*Deprivation quintile*																		
1 (Lowest)	87	336	580							71	272	521						
2	98	370	600	29.5	−13.8, 72.8	0.569	147.7	24.2, 271.1	0.200	73	325	545	44.0	2.0, 86.0	0.621	155.0	1.7, 308.3	1
3	81	314	545	22.5	−18.8, 63.9	0.947	114.3	−0.9, 229.6	0.664	59	294	534	26.5	−12.3, 65.3	0.274	194.0	30.8, 357.2	0.296
4	93	381	588	34.9	−12.4, 82.1	0.375	123.0	2.0, 244.0	0.522	53	282	523	28.0	−14.7, 70.7	0.424	133.0	−17.4, 283.4	0.475
5 (highest)	135	424	616	75.5	7.4, 143.7	0.028	215.0	98.0, 332.0	0.001	83	270	523	42.0	−3.3, 87.3	0.772	163.0	12.0, 314.0	0.797
*Charlson comorbidity*^*b*^																		
0	74	289	542							50	218	481						
1	96	370	588	46.2	1.4, 91.0	0.053	186.3	76.8, 295.9	0.004	130	376	549	110.5	45.6, 175.4	0.002	294.0	137.1, 450.9	<0.001
2	161	400	617	119.9	40.6, 199.2	0.001	200.7	73.3, 328.0	0.010	146	408	598	121.0	29.1, 212.9	0.027	397.0	206.0, 588.0	<0.001
3+	204	504	648	182.0	103.6, 260.5	<0.001	266.7	140.9, 392.4	<0.001	141	473	638	173.5	32.2, 314.8	0.031	366.0	210.6, 521.4	<0.001
*Symptoms*																		
CIBH or RB	64	257	548							50	228	517						
Test anaemia	92	334	557	64.4	25.1, 103.6	<0.001	165.0	52.5, 277.5	0.040	129	367	537	95.5	11.3, 179.7	0.088	259.0	86.0, 432.0	0.014
Non red-flag	159	451	627	147.7	87.5, 207.9	<0.001	272.3	166.3, 378.4	<0.001	128	384	551	111.0	47.0, 175.0	0.002	310.0	158.9, 461.1	<0.001

^a^The reference group corresponds to 55–64 year old men, in the least deprived group, without comorbidities, with change in bowel habit (CIBH) or rectal bleeding (RB).

^b^Charlson comorbidity score.

The multivariable quantile regression analysis showed that increasing comorbidity burden was associated with a significantly longer symptom-to-test interval: for example, for colon cancer patients with Charlson comorbidity score of 2, the adjusted median interval was 120 days [95% CI 40.6; 199.2] versus 30 days [95% CI 1.7; 44.7] among non-comorbid patients with similar symptoms and socio-demographic characteristics (Table 2). Older age and female gender (for colon and rectal cancer, respectively) were also associated with longer intervals, as were anaemia and non red-flag presenting symptoms versus rectal bleeding/change in bowel habit. Similar findings were observed for test-to-diagnosis and symptom-to-diagnosis intervals (Supplementary Tables 1–2).

Sensitivity analyses focusing on patients who underwent bowel endoscopy, irrespective of whether it was the first investigation or rather preceded by other non-specific abdominal/pelvic ultrasound scan, CT or MRI, showed similar findings, with a median symptom-to-endoscopy interval of 179 versus 85 days for comorbid versus non-comorbid patients and significantly longer intervals with increasing Charlson score (Supplementary Table 4).

### Risk of emergency cancer diagnosis by patient characteristics, symptoms, comorbidity burden/status and type of diagnostic investigation

**Fig. 3:**
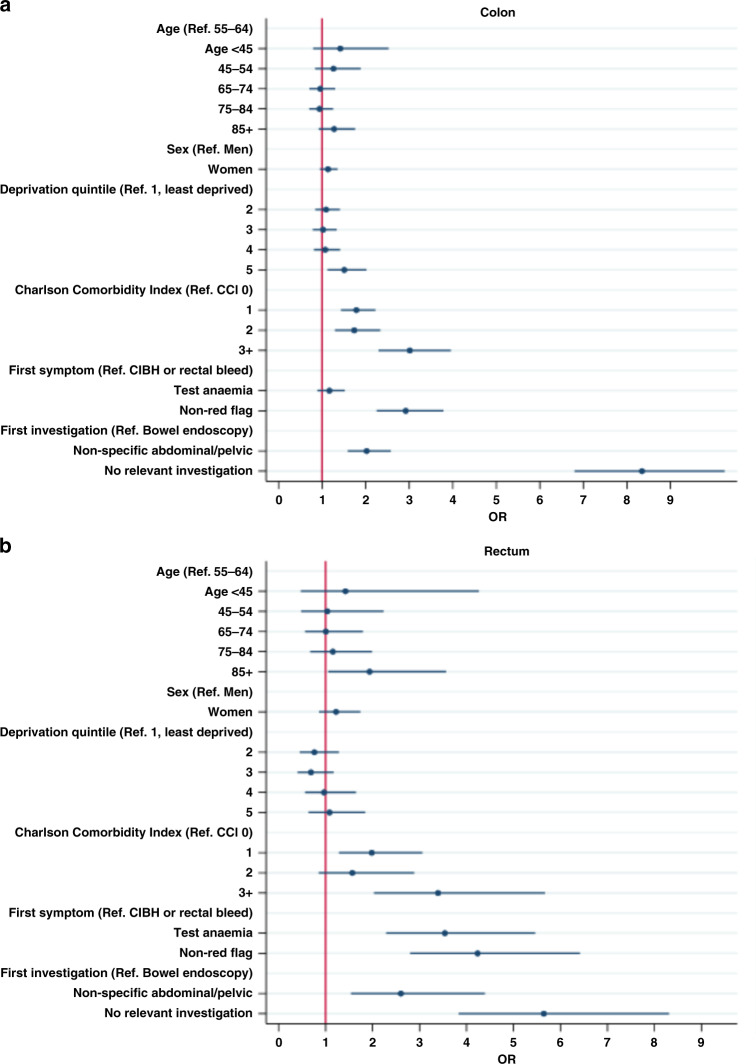
Multivariable logistic regression odds ratios (OR) for the association between patient characteristics, symptoms, Charlson comorbidity index (CCI) score, investigations and emergency cancer diagnosis (colon *n* = 3215; rectum *n* = 1621). **a** Colon cancer. **b** Rectal cancer. CIBH change in bowel habit.

Among colon cancer patients, the proportion of emergency diagnosis increased with increasing Charlson comorbidity score (23%, 35%, 33%, 47% for scores 0, 1, 2, 3+; adjusted (a)OR (vs 0 morbidities) = 1.8 [1.4, 2.2]; 1.7 [1.3, 2.3]; 3.0 [2.3, 4.0]) (Fig. 3 and Supplementary Table 5). The risk of emergency diagnosis was particularly high for patients with common morbidities, such as CVD (43%, aOR = 2.0 [1.5–2.5], and for those with chronic renal disease (44%, aOR = 1.5 [1.0–2.1] (Table 3).

**Table 3:** Associations between the most common specific morbidities recorded in secondary care and the risk of emergency cancer diagnosis: unadjusted and adjusted odds ratios (OR) from multivariable logistic regression^a^.

	Colon cancer					Rectal cancer				
	Emergency diagnosis *n* (%)	Unadjusted OR and 95% CI		Multivariate OR and 95% CI		Emergency diagnosis *n* (%)	Unadjusted OR and 95% CI		Multivariate OR and 95% CI	
*Cardiovascular disease*										
No	705 (26.8)					128 (9.1)				
Yes	252 (43.2)	2.1	1.7, 2.5	2.0	1.5, 2.5	57 (27.3)	2.1	1.7, 2.5	2.7	1.7, 4.3
*Chronic obstructive pulmonary disease*										
No	758 (28.5)					141 (10.0)				
Yes	199 (35.5)	1.4	1.1, 1.7	1.1	0.9, 1.4	44 (20.3)	1.4	1.1, 1.7	1.4	0.9, 2.1
Diabetes										
No	792 (29.1)					153 (10.8)				
Yes	165 (33.5)	1.2	1.0, 1.5	1.2	0.9, 1.5	32 (15.9)	1.2	1.0, 1.5	1.2	0.7, 1.9
*Chronic renal disease*										
No	857 (28.7)					162 (10.6)				
Yes	100 (44.6)	2.0	1.5, 2.7	1.5	1.0, 2.1	23 (25.3)	2.0	1.5, 2.7	1.3	0.7, 2.5

^a^Multivariable models included age, sex, deprivation, symptoms, type of first investigation and specific morbidities.

Patients initially investigated with non-specific abdominal tests (28%; aOR = 2.0 [1.6,2.6]) or without relevant investigations (62%; aOR = 8.3 [6.7, 10.4]) also had a higher risk of emergency diagnosis, compared to those with a colonoscopy/sigmoidoscopy as first test (13%). Having only non red-flag symptoms versus having either rectal bleeding or change in bowel habit increased the risk of emergency diagnosis (aOR = 2.9 [2.2, 3.8]) (Fig. 3 and Supplementary Table 5).

Among rectal cancers, the proportion of emergency diagnosis was lower, but the risk was increased by the same factors as for colon cancer, at a comparable degree; additionally, anaemia was also associated with emergency presentations among rectal cancer patients (aOR = 3.5 [2.3, 5.5]) (Supplementary Table 5).

Using the Charlson score or the morbidity count consistently indicated similar associations between morbidity burden and emergency cancer diagnosis (Supplementary Tables 5–6).

## Discussion

### Summary

Our findings indicate that comorbid individuals often present with anaemia, rather than with localising symptoms, and they are less likely promptly investigated with colonoscopy/sigmoidoscopy. Among patients presenting to primary care with CRC-relevant symptoms, diagnostic intervals before a cancer diagnosis are on average more than twice as long for patients with one or more hospital-recorded comorbidity compared to non-comorbid individuals. The risk of emergency cancer diagnosis increases with increasing comorbidity burden and is particularly high for individuals with CVD compared to those without.

### Interpretation and comparison with the literature

The study highlighted that comorbid versus non-comorbid patients had more frequently records of anaemia, which was associated with a lower likelihood of prompt colonoscopy/sigmoidoscopy and a higher risk of emergency presentation. Previous research indicated that anaemia can be associated with missed opportunities for earlier cancer diagnosis [42, 43]. Our study suggests that this might particularly apply to comorbid patients and it may partially explain their higher risk of emergency cancer diagnosis.

Atypical symptoms are known to be associated with longer diagnostic intervals [44, 45] and an increased risk of emergency diagnosis [42, 46, 47], but evidence is scant on variations in symptomatic presentations by comorbidity status and how this might influence the timely diagnosis of cancer [11]. A previous study indicated that colon cancer patients with ‘serious’ comorbidities often repeatedly presented to their doctor with cancer-related symptoms before an emergency cancer diagnosis [22]. Research based either on primary care [13, 48] or secondary care data [12, 49] reported longer time to cancer diagnosis for comorbid patients [11], but to our knowledge no population-based study has examined variations in the use and timing of endoscopy by patients’ comorbidity status and symptoms.

Our findings indicate not only that symptomatic presentations of CRC might differ by comorbidity status; comorbidities are also strongly associated with a lower likelihood of prompt colonoscopy/sigmoidoscopy and a higher risk of emergency cancer diagnosis, independently of symptoms and socio-demographic characteristics. This suggests that more than one mechanism might be at play in increasing emergency presentations. Hospital-recorded comorbidities are often complex to manage. Thus, despite being associated with frequent healthcare encounters, instead of providing opportunities for earlier cancer diagnosis, they can interfere with prompt investigations of an as-yet undiagnosed cancer [22] through the ‘competing demands’ mechanism, particularly if symptoms are vague. For instance, severe CVD might need to be appropriately managed in some patients before performing invasive investigations, such as colonoscopy, which might prolong the time before cancer diagnosis. This is in line with our finding of a lower likelihood of prompt colonoscopy/sigmoidoscopy in patients with CVD, similar to an American study on missed opportunities for endoscopy in CVD patients [43]. It should be noted that shorter diagnostic intervals might sometimes reflect rapidly progressing aggressive cancers [50, 51]. However, our findings suggest possible opportunities for earlier diagnosis at least in some patients with comorbidities and cancer-related symptoms, who experienced prolonged time before investigations.

### Strengths and limitations

Strengths of the study include the use of real world population-based data, encompassing symptomatic presentations in primary care, hospital-recorded comorbidities, investigations performed in hospital and ‘routes to diagnosis’ information. The study demonstrates the usefulness of integrating clinical data spanning across the entire diagnostic pathway. The methodology, which has highlighted key factors to be considered for enhancing risk-stratification and improve diagnostic timeliness, can be usefully applied to other cancers and patient populations. It might be particularly relevant for lung or upper GI cancers, which are often diagnosed late and frequently present in patients with comorbidities [11]. The use of validated algorithms for defining hospital-recorded comorbidities allowed us to provide novel evidence on the likely role played by severe morbidities in influencing the use and timeliness of colonoscopy.

Using different morbidity markers and measures (Charlson score, morbidity count, presence of specific morbidities) consistently indicated strong associations between morbidity burden, longer diagnostic intervals and emergency cancer diagnosis. Some associations might have appeared significant by chance due to testing for multiple variables. However, the strong evidence (*p*-values < 0.001) for variation by comorbidity burden in diagnostic intervals and emergency diagnosis, suggests that such associations are unlikely due to chance.

The study limitations include the likely underestimation of less severe morbidities typically managed in primary care, for example, well-controlled diabetes or benign gastro-intestinal conditions [16, 52, 53]. Mental health issues have also not been examined here, while they might play an important role in influencing the timely diagnosis of cancer [44, 54]. Our study did not support the hypothesis whereby morbidities requiring regular healthcare contacts (e.g. diabetes) might provide opportunities for earlier cancer diagnosis; nor did it support the ‘alternative explanation’ mechanism whereby morbidities with similar signs/symptoms to CRC can lead to diagnostic delays [11, 13, 17, 44]. We cannot exclude that such effects might apply to comorbidities managed in primary care [44]. Previous research indicated that well-controlled diabetes or hypertension monitoring might sometimes offer opportunities for earlier cancer diagnosis [11, 22]. Examining the role of less sever conditions managed in primary care deserves to be examined in detail in future studies. However, focusing on morbidities severe enough to be recorded during a hospital admission in the 6 years pre-diagnosis has face validity regarding the current study question, as these morbidities may influence clinicians’ decision-making about the use of invasive diagnostic procedures, such as colonoscopy, that may require pre-operative assessment.

Additionally, studies based on patient interviews are needed to evaluate patient-related factors, such as self-management of symptoms or comorbidities, anxiety and/or willingness to undergo invasive investigations, as these factors might also influence the use of investigations and diagnostic timeliness. Moreover, while we accounted for clustering of patients by GP practice, details on area-specific availability of diagnostic services and waiting times could provide further insights into possible reasons for longer diagnostic intervals.

CVD and other conditions found here to be associated with prolonged diagnostic intervals and emergency presentations are aetiologically unrelated to CRC and they probably developed over many years, suggesting that reverse causation unlikely explains our findings. We did not have information on the specific event triggering the emergency admission (bowel perforation, occlusion or a CVD-related emergency). However, irrespective of the triggering event, reducing emergency cancer diagnosis is a key public health target [1, 3], as it is strongly associated with advanced cancer stage and poor survival [7, 55].

We focused on the use of lower GI endoscopies, which are well documented in HES. Having used routinely collected clinical codes, ascertainment bias cannot be excluded. However, previous validation studies have shown 96% accuracy for routine data collected in HES [32, 33]. Imaging tests (including ultrasound scans, CT/MRI) are only partially captured in HES, and imaging data from HES-DID might allow to explore this further [12].

Linked data were available only up to 2015 and more recent data is needed, considering persisting inequalities in emergency presentations and poorer survival in comorbid patients [56, 57].

Our study does not exactly map on the primary and secondary care intervals, as defined in other research [12, 44, 45, 50], since we relied on the date of test performance, but lacked information on the GP referral date. While the symptom-to-test interval in our study is longer than the referral interval reported in a national audit [14], it reflects the time symptomatic patients wait before being investigated.

We lacked information on hereditary non-polyposis CRC or familial adenomatous polyposis, but they likely represent a small minority of cases (<2%) in our study population of mostly older individuals.

### Implications for practice and research

Lower GI endoscopy is the gold standard for investigating patients with symptoms suggestive of CRC. Non-invasive strategies, such as FIT or other promising tools for supporting the diagnostic process in primary care [4–6, 58], combined with appropriate safety-netting (including pre-booked short-term follow-up visits), can be used for selecting patients at increased risk and referring them for urgent investigations [59]. FIT might be particularly useful for patients with serious comorbidities to prioritise those requiring immediate endoscopy and reduce emergency presentations, but risks and benefits will need to be evaluated. Older age (75 years and over) was also significantly associated with longer diagnostic intervals for colon cancer possibly due to the need of performing pre-colonoscopy assessments/preparations to minimize the risks associated with invasive investigations especially in older and frail patients. Research is needed on appropriate testing strategies for comorbid and older individuals and on the role played by frailty and the risks of invasive testing in influencing clinician’s decision-making and patient’s attitudes to testing. Using FIT and minimally invasive radiology tests might be particularly useful as first-line assessment in older and comorbid patients and reduce their higher risk of emergency presentations. Timely access to cancer diagnostic services is essential, especially for symptomatic individuals with multimorbidity, who can be particularly vulnerable. This has been emphasised by the recent Covid-19 crisis, during which many cancer investigations have been postponed with possible dramatic consequences especially for multimorbid cancer patients [18–20, 60].

As patients with multiple conditions might not spontaneously report all symptoms, especially if vague [61], targeted patient education campaigns might be useful for individuals with chronic conditions, as well as more systematic adoption of symptom elicitation and holistic approaches during doctor-patient encounters [62, 63]. Closer interaction and easier access to specialist advice for GPs and use of multi-disciplinary rapid diagnostic centres might be particularly important for comorbid patients [4, 64, 65]. Little is known on how comorbidities might influence CRC screening participation, but some studies suggested a lower screening probability for comorbid individuals [11, 29]. Encouraging age-based screening could be particularly valuable to reduce the risk of emergency and advanced stage cancer diagnosis in individuals with morbidities associated with a higher CRC risk, such as diabetes [11]. While the wider use of screening may allow diagnosing CRC earlier and substantially reduce emergency presentations in the future, currently only 10% of all CRC in England are diagnosed through screening [24]. This highlights the key importance of improving the diagnosis in symptomatic patients.’

Our findings suggest the potential usefulness of morbidity status as a risk-stratifier when assessing the risk of diagnostic delays in symptomatic patients with as-yet undiagnosed cancer. For example, the positive predictive values associated with anaemia or rectal bleeding may vary by morbidity status, given the observed variations in symptomatic presentations. The present research focused on CRC patients, but the methods and findings can inform further work on other cancer sites, for example, lung or upper GI cancers, which are often diagnosed late and frequently present in patients with comorbidities [11, 53]. Moreover, given the greater risk among comorbid patients of cancer being diagnosed as an emergency and at advanced stage, our findings suggest that a lower threshold for investigations might be beneficial, but future studies are warranted aimed at evaluating risks and benefits of different thresholds. Research is also warranted aimed at developing enhanced risk-stratification tools to prioritise patients needing urgent tests, accounting for comorbidity, symptoms and demographic characteristics.

## Conclusions

The study has provided novel evidence on factors contributing to prolonged diagnostic intervals and emergency cancer diagnosis among the large number of cancer patients with pre-existing morbidities. Comorbid individuals with an as-yet undiagnosed colorectal cancer often present with anaemia, rather than with localising symptoms, and they are less often promptly investigated with colonoscopy/sigmoidoscopy. Our findings indicate the potential usefulness of morbidity status, in addition to symptoms, to help prioritize patients needing urgent tests. Enhanced risk-stratification tools, accounting for comorbidity status, should be developed to support clinical decision-making. While risks and benefits of different thresholds for investigations among comorbid individuals should be evaluated, non-invasive testing strategies, such as FIT, might be particularly useful for patients with serious comorbidities and non-localising symptoms to prioritise those requiring endoscopy and reduce emergency presentations.

## Supplementary information

Supplemental Material

## Data Availability

Data used in this study were accessed through the Clinical Practice Research Datalink that is subject to protocol approval by an Independent Scientific Advisory Committee and cannot directly be shared.

## Code availability

Code-lists for relevant symptoms and investigations are available as Supplementary box 1–2.
